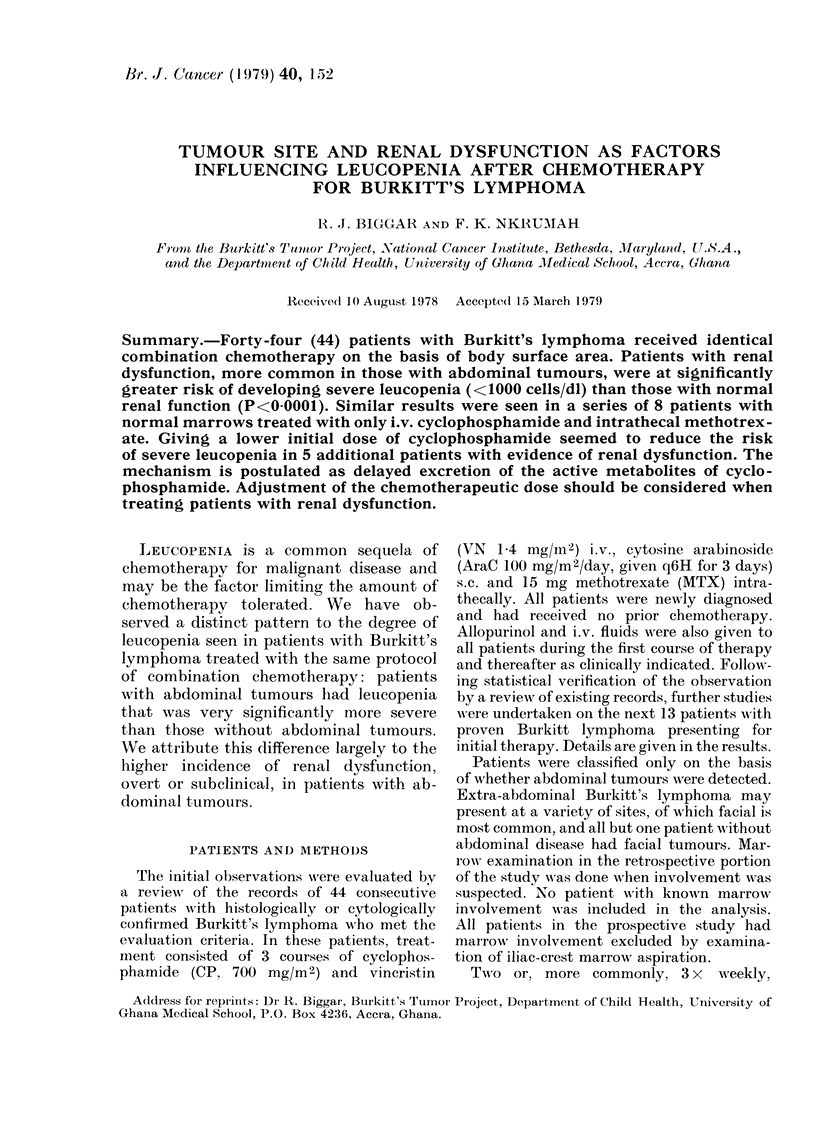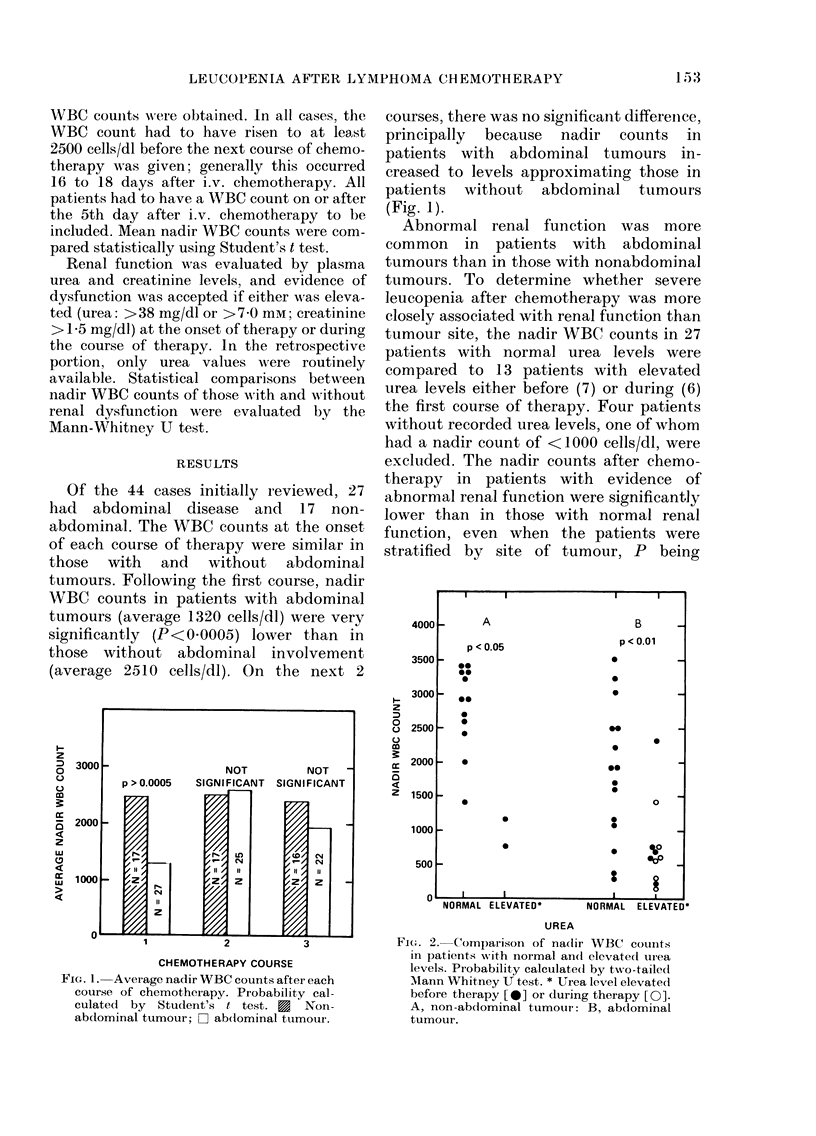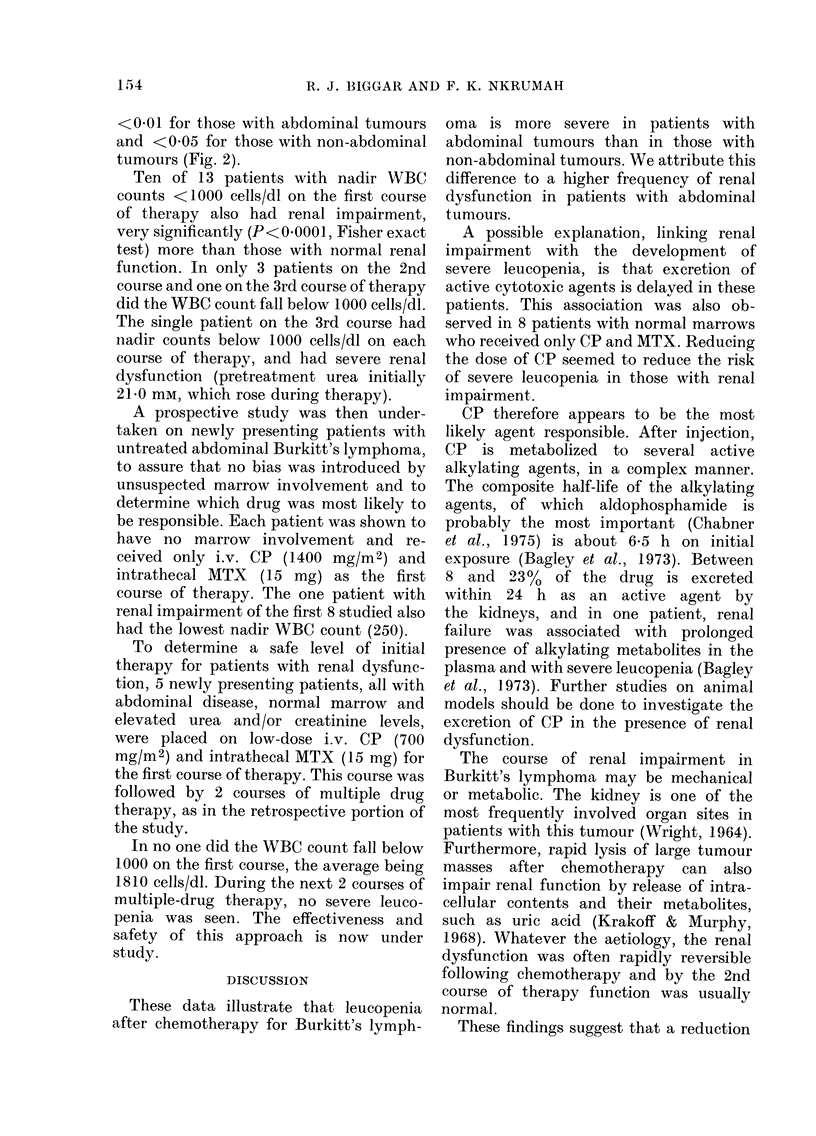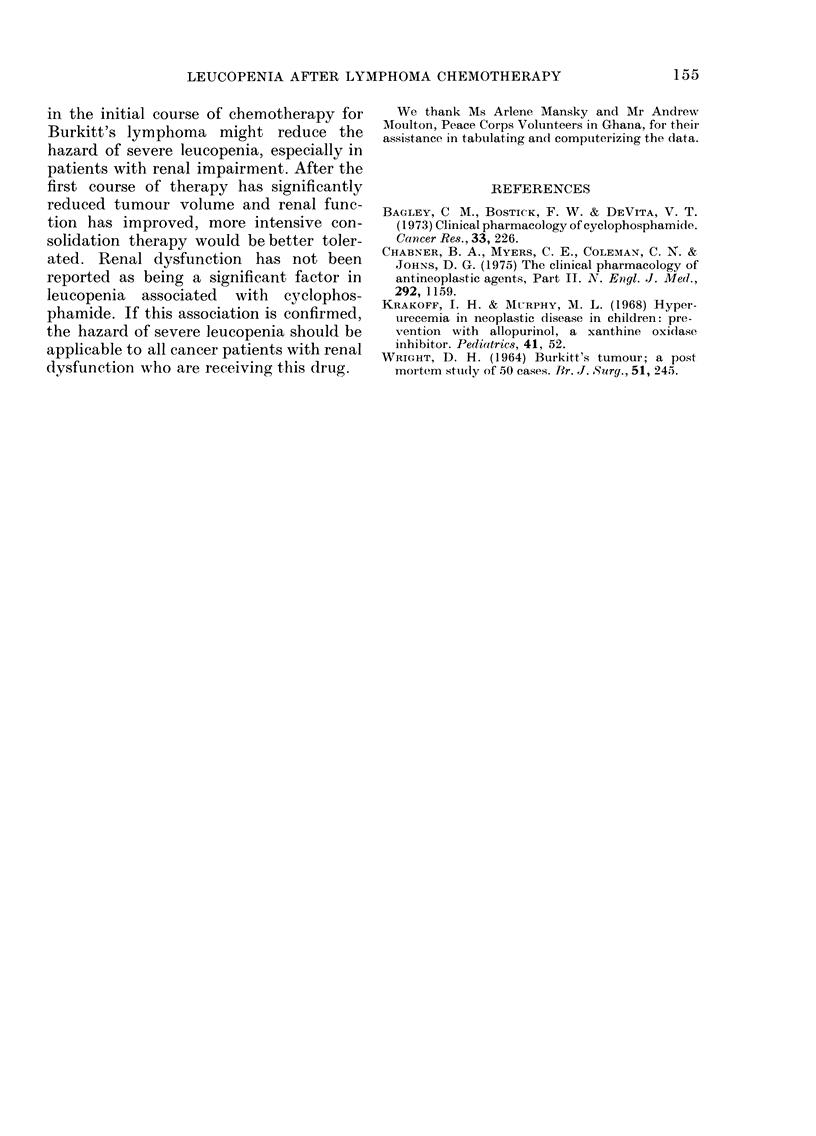# Tumour site and renal dysfunction as factors influencing leucopenia after chemotherapy for Burkitt's lymphoma.

**DOI:** 10.1038/bjc.1979.151

**Published:** 1979-07

**Authors:** R. J. Biggar, F. K. Nkrumah

## Abstract

Forty-four (44) patients with Burkitt's lymphoma received identical combination chemotherapy on the basis of body surface area. Patients with renal dysfunction, more common in those with abdominal tumours, were at significantly greater risk of developing severe leucopenia (less than 1000 cells/dl) than those with normal renal function (P less than 0.0001). Similar results were seen in a series of 8 patients with normal marrows treated with only i.v. cyclophosphamide and intrathecal methotrexate. Giving a lower initial dose of cyclophosphamide seemed to reduce the risk of severe leucopenia in 5 additional patients with evidence of renal dysfunction. The mechanism is postulated as delayed excretion of the active metabolites of cyclophosphamide. Adjustment of the chemotherapeutic dose should be considered when treating patients with renal dysfunction.


*Br. J. Cancer* (1979) **40**, 152

### TUMOUR SITE AND RENAL DYSFUNCTION AS FACTORS INFLUENCING LEUCOPENIA AFTER CHEMOTHERAPY FOR BURKITT'S LYMPHOMA

R. J. BIGGAR AND F. K. NKRUMAH

*From the Burkitt's Tumor Project, National Cancer Institute, Bethesda, Maryland, U.S.A., and the Department of Child Health, University of Ghana Medical School, Accra, Ghana*

Received 10 August 1978 Accepted 15 March 1979

**Summary.—Forty-four (44) patients with Burkitt's lymphoma received identical combination chemotherapy on the basis of body surface area. Patients with renal dysfunction, more common in those with abdominal tumours, were at significantly greater risk of developing severe leucopenia ($<$1000 cells/dl) than those with normal renal function ($P<0{\cdot}0001$). Similar results were seen in a series of 8 patients with normal marrows treated with only i.v. cyclophosphamide and intrathecal methotrexate. Giving a lower initial dose of cyclophosphamide seemed to reduce the risk of severe leucopenia in 5 additional patients with evidence of renal dysfunction. The mechanism is postulated as delayed excretion of the active metabolites of cyclophosphamide. Adjustment of the chemotherapeutic dose should be considered when treating patients with renal dysfunction.**

LEUCOPENIA is a common sequela of chemotherapy for malignant disease and may be the factor limiting the amount of chemotherapy tolerated. We have observed a distinct pattern to the degree of leucopenia seen in patients with Burkitt's lymphoma treated with the same protocol of combination chemotherapy: patients with abdominal tumours had leucopenia that was very significantly more severe than those without abdominal tumours. We attribute this difference largely to the higher incidence of renal dysfunction, overt or subclinical, in patients with abdominal tumours.

### PATIENTS AND METHODS

The initial observations were evaluated by a review of the records of 44 consecutive patients with histologically or cytologically confirmed Burkitt's lymphoma who met the evaluation criteria. In these patients, treatment consisted of 3 courses of cyclophosphamide (CP, 700 mg/m$^2$) and vincristin (VN 1·4 mg/m$^2$) i.v., cytosine arabinoside (AraC 100 mg/m$^2$/day, given q6H for 3 days) s.c. and 15 mg methotrexate (MTX) intrathecally. All patients were newly diagnosed and had received no prior chemotherapy. Allopurinol and i.v. fluids were also given to all patients during the first course of therapy and thereafter as clinically indicated. Following statistical verification of the observation by a review of existing records, further studies were undertaken on the next 13 patients with proven Burkitt lymphoma presenting for initial therapy. Details are given in the results.

Patients were classified only on the basis of whether abdominal tumours were detected. Extra-abdominal Burkitt's lymphoma may present at a variety of sites, of which facial is most common, and all but one patient without abdominal disease had facial tumours. Marrow examination in the retrospective portion of the study was done when involvement was suspected. No patient with known marrow involvement was included in the analysis. All patients in the prospective study had marrow involvement excluded by examination of iliac-crest marrow aspiration.

Two or, more commonly, 3× weekly,

Address for reprints: Dr R. Biggar, Burkitt's Tumor Project, Department of Child Health, University of Ghana Medical School, P.O. Box 4236, Accra, Ghana.

WBC counts were obtained. In all cases, the WBC count had to have risen to at least 2500 cells/dl before the next course of chemotherapy was given; generally this occurred 16 to 18 days after i.v. chemotherapy. All patients had to have a WBC count on or after the 5th day after i.v. chemotherapy to be included. Mean nadir WBC counts were compared statistically using Student's $t$ test.

Renal function was evaluated by plasma urea and creatinine levels, and evidence of dysfunction was accepted if either was elevated (urea: $>38$ mg/dl or $>7{\cdot}0$ mM; creatinine $>1{\cdot}5$ mg/dl) at the onset of therapy or during the course of therapy. In the retrospective portion, only urea values were routinely available. Statistical comparisons between nadir WBC counts of those with and without renal dysfunction were evaluated by the Mann-Whitney U test.

### RESULTS

Of the 44 cases initially reviewed, 27 had abdominal disease and 17 non-abdominal. The WBC counts at the onset of each course of therapy were similar in those with and without abdominal tumours. Following the first course, nadir WBC counts in patients with abdominal tumours (average 1320 cells/dl) were very significantly ($P<0{\cdot}0005$) lower than in those without abdominal involvement (average 2510 cells/dl). On the next 2 courses, there was no significant difference, principally because nadir counts in patients with abdominal tumours increased to levels approximating those in patients without abdominal tumours (Fig. 1).

Abnormal renal function was more common in patients with abdominal tumours than in those with nonabdominal tumours. To determine whether severe leucopenia after chemotherapy was more closely associated with renal function than tumour site, the nadir WBC counts in 27 patients with normal urea levels were compared to 13 patients with elevated urea levels either before (7) or during (6) the first course of therapy. Four patients without recorded urea levels, one of whom had a nadir count of $<1000$ cells/dl, were excluded. The nadir counts after chemotherapy in patients with evidence of abnormal renal function were significantly lower than in those with normal renal function, even when the patients were stratified by site of tumour, $P$ being

Fig. 1.—Average nadir WBC counts after each course of chemotherapy. Probability calculated by Student's $t$ test. ▨ Non-abdominal tumour; □ abdominal tumour.

Fig. 2.—Comparison of nadir WBC counts in patients with normal and elevated urea levels. Probability calculated by two-tailed Mann Whitney U test. * Urea level elevated before therapy [●] or during therapy [○]. A, non-abdominal tumour: B, abdominal tumour.

$<0 \cdot 01$ for those with abdominal tumours and $<0 \cdot 05$ for those with non-abdominal tumours (Fig. 2).

Ten of 13 patients with nadir WBC counts $<1000$ cells/dl on the first course of therapy also had renal impairment, very significantly ($P<0 \cdot 0001$, Fisher exact test) more than those with normal renal function. In only 3 patients on the 2nd course and one on the 3rd course of therapy did the WBC count fall below 1000 cells/dl. The single patient on the 3rd course had nadir counts below 1000 cells/dl on each course of therapy, and had severe renal dysfunction (pretreatment urea initially 21·0 mM, which rose during therapy).

A prospective study was then undertaken on newly presenting patients with untreated abdominal Burkitt's lymphoma, to assure that no bias was introduced by unsuspected marrow involvement and to determine which drug was most likely to be responsible. Each patient was shown to have no marrow involvement and received only i.v. CP (1400 mg/$m^2$) and intrathecal MTX (15 mg) as the first course of therapy. The one patient with renal impairment of the first 8 studied also had the lowest nadir WBC count (250).

To determine a safe level of initial therapy for patients with renal dysfunction, 5 newly presenting patients, all with abdominal disease, normal marrow and elevated urea and/or creatinine levels, were placed on low-dose i.v. CP (700 mg/$m^2$) and intrathecal MTX (15 mg) for the first course of therapy. This course was followed by 2 courses of multiple drug therapy, as in the retrospective portion of the study.

In no one did the WBC count fall below 1000 on the first course, the average being 1810 cells/dl. During the next 2 courses of multiple-drug therapy, no severe leucopenia was seen. The effectiveness and safety of this approach is now under study.

### DISCUSSION

These data illustrate that leucopenia after chemotherapy for Burkitt's lymphoma is more severe in patients with abdominal tumours than in those with non-abdominal tumours. We attribute this difference to a higher frequency of renal dysfunction in patients with abdominal tumours.

A possible explanation, linking renal impairment with the development of severe leucopenia, is that excretion of active cytotoxic agents is delayed in these patients. This association was also observed in 8 patients with normal marrows who received only CP and MTX. Reducing the dose of CP seemed to reduce the risk of severe leucopenia in those with renal impairment.

CP therefore appears to be the most likely agent responsible. After injection, CP is metabolized to several active alkylating agents, in a complex manner. The composite half-life of the alkylating agents, of which aldophosphamide is probably the most important (Chabner *et al.*, 1975) is about 6·5 h on initial exposure (Bagley *et al.*, 1973). Between 8 and 23% of the drug is excreted within 24 h as an active agent by the kidneys, and in one patient, renal failure was associated with prolonged presence of alkylating metabolites in the plasma and with severe leucopenia (Bagley *et al.*, 1973). Further studies on animal models should be done to investigate the excretion of CP in the presence of renal dysfunction.

The course of renal impairment in Burkitt's lymphoma may be mechanical or metabolic. The kidney is one of the most frequently involved organ sites in patients with this tumour (Wright, 1964). Furthermore, rapid lysis of large tumour masses after chemotherapy can also impair renal function by release of intracellular contents and their metabolites, such as uric acid (Krakoff & Murphy, 1968). Whatever the aetiology, the renal dysfunction was often rapidly reversible following chemotherapy and by the 2nd course of therapy function was usually normal.

These findings suggest that a reduction in the initial course of chemotherapy for Burkitt's lymphoma might reduce the hazard of severe leucopenia, especially in patients with renal impairment. After the first course of therapy has significantly reduced tumour volume and renal function has improved, more intensive consolidation therapy would be better tolerated. Renal dysfunction has not been reported as being a significant factor in leucopenia associated with cyclophosphamide. If this association is confirmed, the hazard of severe leucopenia should be applicable to all cancer patients with renal dysfunction who are receiving this drug.

We thank Ms Arlene Mansky and Mr Andrew Moulton, Peace Corps Volunteers in Ghana, for their assistance in tabulating and computerizing the data.

### REFERENCES

BAGLEY, C M., BOSTICK, F. W. & DEVITA, V. T. (1973) Clinical pharmacology of cyclophosphamide. *Cancer Res.*, **33**, 226.

CHABNER, B. A., MYERS, C. E., COLEMAN, C. N. & JOHNS, D. G. (1975) The clinical pharmacology of antineoplastic agents, Part II. *N. Engl. J. Med.*, **292**, 1159.

KRAKOFF, I. H. & MURPHY, M. L. (1968) Hyperurecemia in neoplastic disease in children: prevention with allopurinol, a xanthine oxidase inhibitor. *Pediatrics*, **41**, 52.

WRIGHT, D. H. (1964) Burkitt's tumour; a post mortem study of 50 cases. *Br. J. Surg.*, **51**, 245.